# A person-centered continuity pathway for pediatric contrast-enhanced imaging: sustaining one multisensory context across home, hospital, and scanner transitions

**DOI:** 10.3389/frhs.2026.1940926

**Published:** 2026-09-30

**Authors:** Tommaso Liuzzi, Carmela Napolitano, Fiammetta D’Arienzo, Martina Frascari Diotallevi, Donatella Lettori, Andrea Magistrelli, Aurelio Secinaro

**Affiliations:** 1 Neurorehabilitation Unit, Department of Pediatric Neurorehabilitation, IRCCS Bambino Gesù Children’s Hospital, Rome, Italy; 2 Advanced Cardio-Thoracic, Vascular and Fetal Imaging Unit, IRCCS Bambino Gesù Children’s Hospital, Rome, Italy; 3 Radiodiagnostic Unit Palidoro, IRCCS Bambino Gesù Children’s Hospital, Rome, Italy

**Keywords:** care pathway, Euterpe method, music therapy, nonpharmacological intervention, pediatric imaging, person-centered care, procedural preparation, quality improvement

## Abstract

**Background:**

Pediatric contrast-enhanced computed tomography (CT) and magnetic resonance imaging (MRI) require sustained cooperation, procedural immobility, and venous access, creating operational and experiential challenges for children, families, and imaging services. Existing nonpharmacological interventions generally address isolated procedural segments and do not preserve a familiar context across home, hospital, and scanner transitions.

**Innovation:**

Euterpe-Radiology (Euterpe-RAD) is a consent-gated, five-module institutional pathway that sustains one autobiographically anchored multisensory context—family-voice recordings, a personalized therapeutic composition, and child- or family-selected visual content—from home familiarization through hospital preparation, venous access, acquisition, and closure. Continuity is defined by content identity, coverage of four predefined transitions, and stability of the caregiver and therapeutic roles. The added value of continuity over single-segment preparation is a design hypothesis and is not tested here.

**Approach:**

This community case study describes pathway design and initial implementation under institutional quality-improvement governance. The pathway was offered to eight caregivers; six consented and two declined, both citing the organizational burden of the home-based component. Three children entered Module 1. One completed all five modules and underwent contrast-enhanced CT without sedation. Of the other two, one was excluded when photosensitivity was identified during the opening tele-anamnesis, and one completed home familiarization but did not undergo CT because cannulation was not tolerated. In three additional consenting children, the interval between referring-unit authorization and the scheduled examination was shorter than the 5–7-day home-familiarization window. Module 1 therefore could not be delivered; these children received hospital-stage music-therapy support only and underwent CT without sedation. These encounters are classified as partial deliveries rather than five-module completions. No MRI examination was delivered.

**Conclusions:**

One complete CT pathway was delivered without modification of acquisition or contrast protocols. The exclusions, procedural discontinuation, and partial deliveries define implementation boundaries rather than effects. These counts do not establish effectiveness, acceptability, or a sedation-sparing effect; the MRI branch remains a design specification, and prospective comparative evaluation is required.

## 1 Introduction: nature of the problem and rationale for the proposed innovation

Sustained immobility during magnetic resonance imaging (MRI) acquisition and venous access preceding contrast-enhanced computed tomography (CT) may exceed the spontaneous behavioral capacity of many preschool and some school-age children. Sedation or general anesthesia may therefore be required, with attendant demands for preprocedural evaluation, monitoring, and recovery (1, 2). The implications of early anesthetic exposure for the developing brain remain under scrutiny, while sedated imaging also increases cost, scheduling complexity, and resource use (1–3).

Nonpharmacological strategies include audiovisual distraction, mock-scanner rehearsal, child-life support, parental presence, and immersive virtual reality. Reported effects are largest in children aged approximately 3–10 years, the range in which these interventions have been most extensively evaluated, although evidence quality varies (4). Quality-improvement programs have reduced sedation under routine conditions (5). Parental presence may increase awake MRI completion (6), immersive virtual reality may reduce distress during venipuncture (7), and child-life involvement may reduce anesthesia use (8). These approaches nevertheless tend to operate within a single procedural segment. Preparation achieved at home may be lost at hospital arrival or scanner entry, creating discontinuity across settings and personnel.

Euterpe-Radiology (Euterpe-RAD) was designed around this discontinuity. Rather than adding another isolated preparatory episode, it organizes procedural support around the child's autobiographical context and engages the caregiver as a prepared partner in care, consistent with person- and family-centered practice (9, 10). The pathway extends a previously described multisensory music-therapy method (11) into pediatric imaging while leaving diagnostic acquisition, contrast administration, and departmental safety requirements unchanged. Whether preserved continuity confers benefit beyond a well-executed single-segment intervention is a design hypothesis rather than an established finding. To our knowledge, no published study has isolated the contribution of cross-setting continuity from that of its constituent components, and the present report does not test this hypothesis. Continuity is accordingly specified as a set of verifiable delivery conditions, not as a demonstrated mechanism.

Continuity is defined operationally by three conditions. Content identity requires the same prespecified stimulus set in every module that uses personalized content; a change of delivery channel is permitted, whereas an unauthorized content change is a deviation. Transition coverage requires intentional re-establishment of at least one element of the personalized frame at four transitions: home familiarization to hospital arrival, anteroom briefing to the immersive room, preprocedural immersion to venous access and acquisition, and acquisition to postprocedural closure. Continuous stimulation between scheduled sessions is not required. Relational stability requires continuity of the caregiver role and therapeutic delivery plan; neither the same individual therapist nor the therapist's physical presence beside the gantry is required, although substitutions are documented as deviations. These conditions map to informational, management, and relational dimensions of continuity of care (12) without attributing the Euterpe-RAD model itself to that framework.

The implementation question is whether this personalized frame can be delivered across home, hospital, and scanner transitions under routine service conditions. Whether the pathway changes sedation use, diagnostic image quality, workflow, or procedural experience is a separate effectiveness question not addressed by this report.

## 2 Context: setting and population

### 2.1 Setting, quality-improvement governance, and data protection

Euterpe-RAD operates at IRCCS Bambino Gesù Children's Hospital, a tertiary pediatric referral center in Rome, Italy. The pathway is authorized for cardiac, body, and neuroradiologic indications; initial delivery involved cardiac contrast-enhanced CT for organizational reasons. It is listed in the 2026 institutional Good Practice program in Medical Directorate note no. 661/26, dated March 20, 2026, under patient safety, continuity of care, innovation in patient and family information, health-technology innovation, and resource management. This designation establishes institutional implementation status, not clinical effectiveness.

Three activities remain distinct. Good Practice delivery is routine care conducted by the participating clinical units and documented in institutional health records. The quality-improvement audit examines aggregate service indicators from administrative and technical records, with quarterly review by those units and the clinical area directorate. A prospective study, currently being prepared for research ethics review, will be the only activity to generate research data; records produced before approval will not be retrospectively incorporated into that study.

Selected Standards for QUality Improvement Reporting Excellence 2.0 (SQUIRE 2.0) items (13) were used only as an ancillary reporting aid; no claim of adherence or conformity is made. Before deployment, the Good Practice submission specified an institutional clinical objective of avoiding sedation or general anesthesia whenever clinically and safely feasible, a 6-month baseline, and indicator-specific data sources: anesthesia and scheduling records for sedation use, technical acquisition logs for motion-related sequence repetition, and the radiology information system for the door-in-to-door-out interval. The audit plan stratifies indicators by age, venous-access requirement, and examination type, and records corrective adjustments as version changes. Arousal modulation, reduced movement, and improved cooperation are institutional improvement aims rather than research endpoints; no aggregate indicator value is reported here. The program operates as audit and feedback against that baseline; it did not use a formal improvement-cycle methodology or statistical process control. A structured caregiver satisfaction form is administered as part of routine program documentation and included in the audit; consistent with the reporting boundary stated above, no value is reported in this article.

Personalized materials—including family-voice recordings, images and videos of the child and family, and caregiver video feedback from home familiarization—contain personal data relating to the child and identifiable family members. These data are processed under Regulation (EU) 2016/679 and the Italian Data Protection Code (Legislative Decree 196/2003, as amended by Legislative Decree 101/2018). A single institutional consent document covers pathway delivery and the production, storage, and retention of these materials, with age-appropriate assent. Files are transmitted through the institutional tele-delivery platform, stored in an access-restricted, traceable archive under institutional records-management requirements, and not reused outside the pathway without additional consent.

### 2.2 Population and standard pathway

The pathway includes children aged 4–10 years scheduled for contrast-enhanced CT or MRI, a narrower range than that of the institutional program. Because all eligible examinations require contrast, venous access is planned for each child; departmental contrast procedures remain unchanged. These age limits are operating criteria, not validated developmental thresholds. The lower limit reflects the comprehension and sustained receptive engagement required for the guided narrative and multiday home familiarization of Module 1; the participating teams judged that caregivers could not support these requirements equivalently below approximately 4 years. The upper limit reflects the declining incremental value of the pathway when standard preparation supports awake acquisition. The 4–10-year window does not coincide with the 3–10-year range in which nonpharmacological imaging interventions have been most extensively studied (4, 8). The lower bound is raised for the reason stated above, and no age-dependent effect of Euterpe-RAD is implied. Children unable to maintain the required immobility receive sedation or general anesthesia when clinically indicated; Euterpe-RAD is offered only to the consenting, eligible subgroup, and all others receive standard care.

## 3 Key programmatic elements

### 3.1 Eligibility, consent, and delivery team

Following caregiver consent and documented authorization by the referring unit, eligibility is confirmed during the tele-anamnesis that opens Module 1, before content production. Exclusion criteria are documented, reported, or clinically observed photosensitivity; visual or auditory impairment that precludes use of the multisensory content; insufficient language comprehension for the guided narrative; a clinical indication for sedation or general anesthesia independent of cooperation; contraindications to MRI or contrast administration under departmental protocols; facial skin or infectious conditions incompatible with headset use; or clinical instability that makes prolonged preprocedural preparation inadvisable. Children who are ineligible or whose caregivers decline receive standard care.

The neurorehabilitation team conducts sensory and neuropsychological profiling and produces the personalized therapeutic content from authorized child and family materials (11). Caregivers deliver home familiarization under remote supervision. In the hospital, a qualified music therapist re-establishes the personalized context and coordinates delivery with nursing and radiology staff. A psychotherapist is present throughout pathway delivery, from the opening tele-anamnesis and remote supervision of home familiarization to postprocedural closure, and supports both child and caregiver in every module. Relational stability is defined at the level of the role and the delivery plan rather than of the individual practitioner, and substitutions are documented as deviations. This allocation of roles is consistent with multidisciplinary pediatric imaging support (8, 14).

Planned fidelity measures are home adherence (at least 80% of scheduled administrations per child), traceability of every personalized element to the sensory profile or authorized material, inter-coder agreement for structured content coding, and content-identity and transition-coverage ratios. The draft prospective protocol proposes an intraclass correlation coefficient (ICC) threshold of at least 0.80 for inter-coder agreement; this is a planned criterion, not an observed property of the present deployment, and will be considered prespecified only after the protocol and statistical analysis plan are dated, approved, and prospectively registered. During the reported deployment, the module checklist was completed for each pathway module actually delivered, and substitutions, deviations, and transition coverage were documented as applicable. Section 3.4 reports only the descriptive checklist findings for the single complete pathway; no aggregate fidelity estimate is reported.

### 3.2 The immersive environment: configuration and pre-arrival setup

The immersive environment is the technical condition of content identity. The scenario selected during profiling and administered at home—an underwater, outer-space, or domestic scene—is reproduced in the room, so that the child encounters the same familiar frame on arrival. An environment that is thematic but not identical does not satisfy the first transition.

In the CT branch the acquisition room is configured as the immersive environment, so that preprocedural immersion, venous access, contrast administration, and acquisition occur in one room without transfer of the child. Caregiver briefing takes place in a separate anteroom, where no clinical procedure and no child-directed personalized content are delivered. Caregiver presence follows radiation-protection requirements; acquisition parameters, dose-reduction procedures, and contrast protocols remain unchanged.

The configuration comprises three fixed video projectors, one directed at the ceiling to cover the visual field of the supine child; a portable projector that relays content into the bore when the headset is declined or not tolerated, constituting a permitted change of delivery channel but not of content; a four-loudspeaker system driven by a dedicated computer; and perimeter LED lighting around the scanner. The loudspeaker system is configured to deliver the personalized therapeutic composition and environmental soundscape according to the acoustic specification reported in Supplementary Material 1 (15–17); ear-level output in the delivered CT configuration was not instrumentally verified during the reported deployment. The child is given a soft handheld light element whose intensity the child controls directly, providing a tangible surrogate for caregiver contact when in-room presence is not permitted. It is retained during acquisition only when outside the scan field of view and remains with the child afterward; it is therefore not shared between patients.

The room is prepared one hour before the scheduled examination by the music therapist with nursing and support staff, who load the finalized personalized content and verify audio, video, and projection alignment using a standardized pre-arrival checklist before the child enters the room. The standard configuration is restored at the end of the examination list. All items introduced into the acquisition room were approved by the radiology technical staff for material composition and construction.

In the MRI branch, this equipment does not enter the magnet room. Preprocedural immersion and venous access occur in the immersive room, after which the child is transferred to the magnet room; the standalone headset is not labeled magnetic resonance (MR) Safe or MR Conditional and remains outside the MR controlled access area. Because no MRI examination had been delivered by the census date, this branch remains a design specification. Familiar content would be delivered beneath mandatory hearing protection (18) through an appropriately labeled MR Safe or MR Conditional pneumatic headphone system and an in-bore visual channel controlled from outside the scanner room. These are functional requirements, not a verified scanner-specific installation. The standalone headset operates offline with cloud synchronization disabled; between patients the facial interface and straps are disinfected according to manufacturer and institutional infection-prevention procedures, a single-use face cover is applied, alcohol-based products are avoided on the lenses, hand hygiene precedes and follows fitting, and patient-specific content is removed from the device after each session.

### 3.3 The five modules

#### 3.3.1 Preliminary step: activation

Full-pathway delivery requires a scheduled contrast-enhanced CT or MRI examination, with venous access therefore planned; signed caregiver consent for pathway delivery and audiovisual-data handling; documented electronic authorization from the referring unit for the music therapist to contact the family; and an interval between authorization and the scheduled examination sufficient to accommodate the 5–7-day home-familiarization window. When that interval is unavailable, Module 1 does not begin. Hospital-stage music-therapy support may nevertheless be provided, but the encounter is recorded as a partial delivery rather than as a protocol deviation or five-module completion. When the interval requirement is met, receipt of the referring-unit authorization initiates Module 1. Each module has a standardized delivery checklist specifying entry criteria, required elements, safety gates, and exit criteria. A checklist is completed for each pathway module actually delivered, through the point at which the child leaves the pathway, retained in the institutional record, and used as the source document for the continuity and fidelity indicators defined in Supplementary Material 1.

#### 3.3.2 Module 1: profiling, production, and home familiarizationz

During the tele-anamnesis, the music therapist obtains the child's sensory, affective, and autobiographical profile and confirms eligibility. Identification of an exclusion criterion stops the module and returns the child to standard care. The caregiver provides authorized photographs, videos, family-voice recordings, musical preferences, and other relevant autobiographical material. The therapist produces a personalized therapeutic composition of approximately 3–4 min and integrates it with the visual and auditory material. Each element must be traceable to the profile or authorized source; modifications are documented. The complete audiovisual sequence is administered at home 2–3 times daily for 5–7 days without headset use. Caregiver video feedback is used to verify delivery and tolerability, not to measure effect. Progression requires confirmed eligibility, completed familiarization, and technical availability of the content. Reporting of the music-based component follows the reporting guidelines for music-based interventions (RG-MBI) framework (19), with full specifications in Supplementary Material 1.

#### 3.3.3 Module 2: hospital briefing of the caregiver

On day 0, after arrival at the imaging service, the caregiver is briefed in the designated anteroom. Staff confirm identity, the scheduled examination, continued eligibility, and the absence of relevant clinical change; home delivery and tolerability are reviewed with the caregiver. Staff explain room entry, headset use, venous access, contrast administration, acquisition, and closure, and prepare the caregiver to support co-regulation through consistent verbal contact and, when permitted, noncoercive reassuring touch. All contact remains subject to staff direction and modality-specific safety requirements. No clinical procedure or child-directed personalized content is delivered in the anteroom. The child is then invited into the immersive room.

#### 3.3.4 Module 3: preprocedural immersion

In the immersive room, the familiarized audiovisual sequence is re-established without introducing content foreign to the child's profile. In the CT branch, the headset is fitted before venous access; fit, comfort, communication, clinical observation, and access to the venous site are verified during a 5–15-min preprocedural session. Exposure is stopped for nausea, dizziness, discomfort, visual disturbance, disorientation, or at the child's request, with onset, intensity, and resolution documented by operator and child report. Operator-rated readiness to proceed without sedation is a clinical gate, not an outcome measure. The headset component is not delivered in the MRI branch.

Because the consumer headset is used under continuous clinical supervision in children below the manufacturer's recommended consumer age, and pediatric evidence has not established safe exposure parameters (20, 21), symptoms and total headset exposure from fitting to removal are recorded. The 5–15-min interval specifies the preprocedural immersion session only; any continuation of headset exposure through venous access, contrast administration, positioning, or acquisition in Module 4 is documented within the total exposure time. Device handling, hygiene, and content removal are specified in Section 3.2. No formal institutional risk assessment of this use had been completed by the census date. A structured cooperation score is planned for prospective evaluation but is not in routine use.

#### 3.3.5 Module 4: venous access, contrast administration, and acquisition

In the CT branch, venous access is established in the immersive room while the headset is retained. The headset and environmental content may continue through positioning and acquisition only if they do not compromise the procedure, communication, clinical observation, the anatomical region examined, the scan field of view, diagnostic quality, monitoring, or safety. Once preparation is complete, the music therapist leaves the room while the programmed content continues. Before contrast injection, the caregiver explains the possible transient warmth in clear, nondeceptive language within the familiar superhero-transformation narrative and, when authorized, uses verbal or noncoercive physical reassurance to support stillness. The caregiver leaves before radiation exposure when in-room presence is not permitted. Standard contrast, acquisition, dose-reduction, and radiation-protection procedures remain unchanged; only the supportive environment is extended into acquisition (22).

In the MRI branch, venous access is established in the immersive room before transfer to the magnet room. The headset remains outside the MR controlled access area; familiar content would be conveyed beneath mandatory hearing protection through an appropriately labeled MR Safe or MR Conditional pneumatic headphone system and an in-bore visual channel. Venous-access completion, contrast administration, headset retention or removal, examination completion, interruptions, repeated sequences, and sedation are documented separately.

#### 3.3.6 Module 5: de-immersion and closure

After imaging and contrast administration are completed, any retained headset is removed before the environmental audiovisual content is progressively attenuated. The child is reoriented and receives an age-appropriate explanation of the completed procedure. Immediate observation records residual symptoms and events associated with immersive exposure, venous access, or contrast administration, together with module completion, tolerability, deviations, and any use of sedation. Patient-specific content is removed from temporary systems, authorized files are retained under institutional governance, and devices are sanitized.

Table 1 summarizes module-specific materials, safeguards, and planned measures; Figures 1,2 depict the operational sequence and continuity model; and Supplementary Material 1 provides the full template for intervention description and replication (TIDieR) and RG-MBI specifications (19, 23).

**Table 1:** Module-specific settings, materials, safeguards, and proposed prospective measures. Available service-activity counts are descriptive and are not analyzed as study outcomes. A psychotherapist is present across all five modules.

Module	Setting	Materials	Safety and implementation notes	Proposed prospective measures—not analyzed as outcomes here
1. Profiling, production, and home familiarization	Home; days −7 to −1 (5–7 days)	Tele-anamnesis; family-voice recordings; personalized therapeutic composition (approximately 3–4 min); authorized autobiographical images and video; institutional tele-delivery platform	No headset is used at home. Delivery is caregiver-mediated. Eligibility is confirmed during the tele-anamnesis; identification of an exclusion criterion stops the module and returns the child to standard care.	Home adherence (completed/scheduled administrations; proposed protocol threshold of at least 80% per child); time from consent to eligibility confirmation
2. Hospital briefing of the caregiver	Designated anteroom, day 0, before entry to the immersive room	Structured briefing on the procedure and the caregiver’s co-regulation and continuity-support roles	No clinical procedure, device exposure, or child-directed personalized content. Caregiver presence and contact remain subject to staff direction and modality-specific safety requirements.	Caregiver-reported confidence in performing the role
3. Preprocedural immersion	Immersive room, day 0; in the CT branch, this is the acquisition room configured as the immersive environment	Environmental audiovisual projection; standalone VR headset (Meta Quest 3), operated offline; 5–15-min preprocedural session. The headset component is not delivered in the MRI branch.	The standalone headset is not labeled MR Safe or MR Conditional and remains outside the MR controlled access area. Between patients, the facial interface and straps are disinfected according to manufacturer and institutional procedures; a single-use face cover is applied; alcohol-based products are avoided on the lenses; and hand hygiene precedes and follows fitting. Patient-specific content is removed after use. Exposure is stopped for nausea, dizziness, discomfort, visual disturbance, disorientation, or at the child’s request; symptoms and total headset exposure from fitting to removal are documented, with the 5–15-min preprocedural session distinguished from any continuation through venous access, contrast administration, positioning, or acquisition. When used below the manufacturer's recommended consumer age, the device is operated under continuous clinical supervision; no formal institutional risk assessment had been completed by the census date.	Age-appropriate validated cooperation measure(s) to be prespecified for research evaluation; headset exposure duration and prespecified exposure-related symptoms or adverse events; completion of the headset risk-assessment specification (Section 4.5.3)
4. Venous access, contrast administration, and acquisition	Day 0. CT: immersive/acquisition room, without transfer. MRI: venous access in the immersive room, followed by transfer to the magnet room	CT: headset and environmental content retained through venous access and acquisition when compatible. MRI: appropriately labeled MR Safe or MR Conditional pneumatic headphones and an in-bore visual channel, specified as functional requirements rather than a verified installation	CT acquisition parameters, dose-reduction procedures, ALARA principles, contrast protocols, and routine dose documentation remain unchanged. Caregiver presence follows radiation-protection requirements. Headset retention requires compatibility with anatomy, positioning, scan field of view, diagnostic quality, monitoring, and safety. In MRI, the standalone headset remains outside the MR controlled access area; MR screening, hearing protection, contrast administration, and acquisition parameters remain unchanged. Acoustic output at the child’s head position in the delivered CT configuration was not instrumentally verified during the reported deployment.	Examination completion under pathway conditions, with the analytic denominator fixed at consent and the implementation denominator at the offer; venous-access completion recorded separately from readiness and examination completion; diagnostic image quality by blinded review; door-in-to-door-out interval; sedation-related measures; instrumental verification of acoustic output at the child’s head position under the configured delivery conditions
5. De-immersion and closure	Immersive room or recovery area, day 0	Headset removal where retained; progressive attenuation of environmental audiovisual content; guided reorientation; age-appropriate explanation of the completed procedure	Immediate observation for symptoms or events associated with immersive exposure, venous access, or contrast administration. Patient-specific content is removed from temporary systems; authorized files are retained under institutional governance; devices are sanitized.	Validated child- and caregiver-reported procedural-experience measures selected for the study age range

ALARA, as low as reasonably achievable; CT, computed tomography; MR, magnetic resonance; MRI, magnetic resonance imaging; VR, virtual reality.

**Figure 1:**
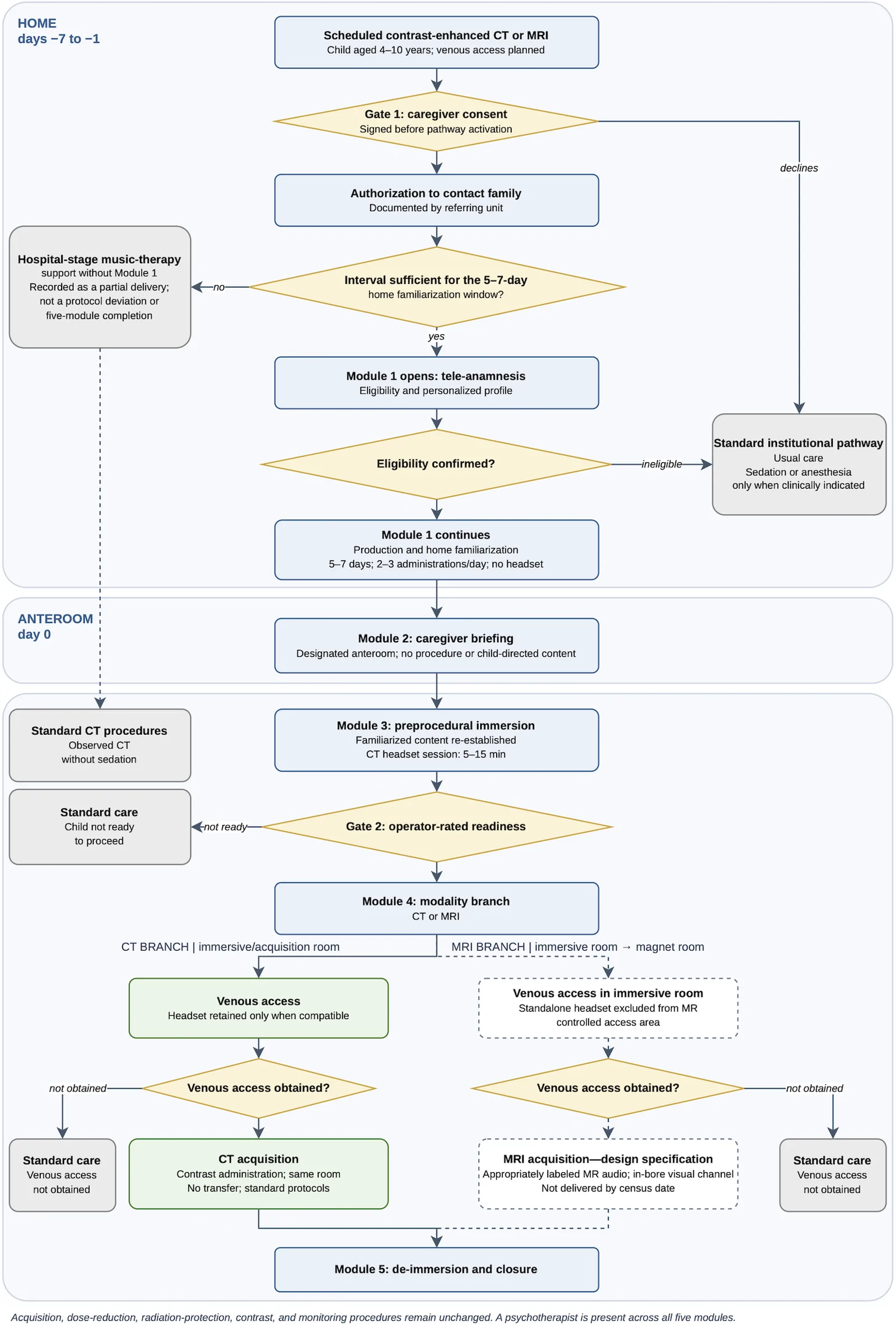
Euterpe–RAD operational pathway. The diagram shows the settings, five modules, process checks governing pathway progression, and the CT and MRI branches. Full-pathway delivery begins with Module 1 after caregiver consent and documented authorization from the referring unit and requires an interval sufficient for the 5–7-day home-familiarization window. When that interval is unavailable, Module 1 does not begin; any hospital-stage music-therapy support is represented separately and classified as a partial delivery rather than as a protocol deviation or five-module completion. Eligibility is confirmed during the tele-anamnesis before content production. Operator-rated readiness precedes Module 4, whereas venous-access completion is recorded as a separate process state. In CT, venous access, contrast administration, and acquisition occur in the immersive/acquisition room without transfer. In MRI, venous access occurs in the immersive room before transfer to the magnet room, and the standalone headset remains outside the MR controlled access area. Dashed MRI elements indicate design specifications not delivered by the census date. Both full-pathway imaging branches proceed to Module 5; full-pathway completion requires documentation of all five modules. Standard acquisition, dose-reduction, radiation-protection, contrast, and monitoring procedures remain unchanged.

**Figure 2:**
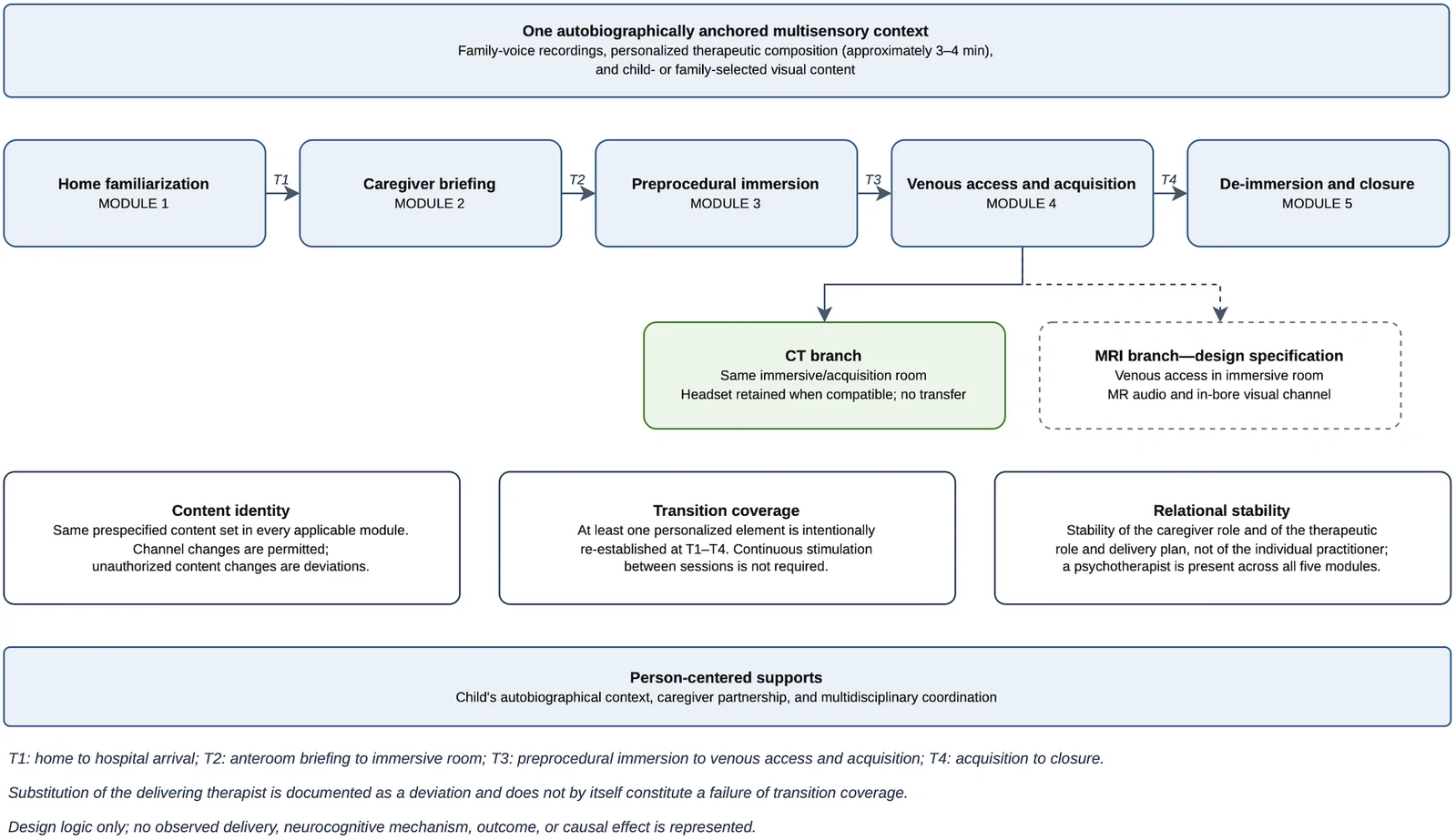
Operational definition of continuity. One autobiographically anchored multisensory context—family-voice recordings, the personalized therapeutic composition, and child- or family-selected visual content—is maintained across the five modules. Continuity comprises content identity, coverage of transitions T1–T4, and stability of caregiver involvement and the therapeutic role and delivery plan. T1 links home familiarization to hospital arrival; T2, anteroom briefing to the immersive room; T3, preprocedural immersion to venous access and acquisition; and T4, acquisition to postprocedural closure. Transition coverage requires intentional re-establishment of the personalized context, not continuous stimulation. The same therapist need not remain present throughout or beside the gantry; substitutions are documented as deviations. CT and MRI use different delivery channels in Module 4. The figure represents the pathway’s design logic, not observed delivery, a neurocognitive mechanism, an outcome, or a causal effect.

### 3.4 Implementation status

Operational delivery under Euterpe-RAD v2.0 began in mid-April 2026. Because the service remains active, counts are reported through the prespecified census date of July 16, 2026.

The pathway was offered to eight caregivers. Six consented and two declined, both citing the organizational burden of the home-based component on the family. Three of the six consenting children entered Module 1. One completed all five modules and underwent contrast-enhanced CT without sedation; in this single complete pathway, all four predefined transitions were documented as covered on the module checklist and no content deviation was recorded, although no aggregate indicator value is derivable from one delivery. One child was excluded when photosensitivity was identified during the opening tele-anamnesis and returned to standard care. One completed Module 1 and entered the immersive room but did not undergo CT because venous access could not be established when cannulation was not tolerated; this was classified as a procedural discontinuation, distinct from the readiness assessment.

In the remaining three consenting children, the interval between referring-unit authorization and the scheduled examination was shorter than the 5–7-day home-familiarization window. Module 1 therefore could not be delivered. These children received hospital-stage music-therapy support only and underwent CT without sedation. Because Module 1 establishes the personalized context on which content identity and coverage of the first transition depend, these encounters were classified as partial deliveries rather than five-module completions.

Thus, four of the six consenting children underwent CT without sedation: one after the complete pathway and three after partial delivery. No MRI examination was delivered. These counts describe implementation, not effectiveness; they support neither attribution of sedation status to the pathway nor inference about acceptability.

Over the reporting interval, the participating cardiac imaging unit performed a routine volume of approximately six cardiac CT and six cardiac MRI examinations per week, corresponding to approximately 155 examinations across the thirteen weeks to the census date. This service-volume figure was not filtered by age band or contrast use and therefore represents an upper bound on the pool of potentially eligible examinations, within which the pathway was offered to eight caregivers. Referrals were not screened prospectively against pathway criteria, and the clinical decision to offer the pathway was not documented; consequently, the number of children considered and the reasons for non-offer could not be reconstructed. Accrual reflected clinical referral and radiology scheduling. The eight offers and six consents therefore constitute a clinician-selected subset of unspecified representativeness: delivery is demonstrated within that subset and does not establish executability under consecutive offering. Neurobehavioral stratification modifies pathway intensity and is not an exclusion criterion. No comparator, aggregate fidelity estimate, blinded image-quality assessment, effectiveness outcome, or statistical analysis is reported.

## 4 Discussion: practical implications and lessons learned

### 4.1 Relation to existing interventions

A randomized trial compared generic virtual reality (VR)-based preparation, a standard manual, and a child-life program in 92 children aged 4–13 years before a simulated head scan, with no clinically significant between-group differences in scan success or child-reported anxiety (24). Euterpe-RAD differs operationally: its content is autobiographical and is retained from home familiarization through preprocedural and procedural delivery. Whether this continuity improves outcomes relative to single-phase preparation or usual care remains untested and requires a comparator. The pathway is complementary to technical strategies such as abbreviated and standardized acquisition protocols (25).

### 4.2 Person-centered operationalization and the multidisciplinary team

Euterpe-RAD is a pathway design rather than a new device or acquisition technique. Maintaining the same personalized context across settings and personnel depends on multidisciplinary coordination (8, 14). Child-derived autobiographical material, caregiver partnership, and supported participation operationalize principles of person-centered practice and healthcare coproduction (26, 27). The potentially transferable element is this pathway logic—personalized continuity, caregiver-mediated preparation, modality-specific delivery, safety gating, and fallback to usual care—rather than the local configuration. Implementation elsewhere would depend on staffing, equipment, and governance; replication has not been tested.

### 4.3 Lessons learned from initial delivery

Initial delivery identified three implementation considerations.

Identification of photosensitivity during the tele-anamnesis illustrates why eligibility should be resolved before content production, home familiarization, or device exposure. The planned prospective protocol therefore includes an explicit photosensitivity item in the opening profile.

In one child, tolerance of the immersive environment did not extend to cannulation. Venous-access completion must consequently be recorded separately from operator-rated readiness and examination completion.

Hospital-stage music-therapy support delivered without Module 1 cannot be classified as full-pathway delivery because the personalized context has not been established through home familiarization. Full-pathway completion therefore requires documentation of all five modules. In all three partial deliveries, Module 1 was omitted because the interval between referring-unit authorization and the scheduled examination was shorter than the familiarization window. Lead time constrained these three encounters; three instances cannot establish whether it is the principal constraint on full-pathway delivery. Sufficient lead time is now stated as a requirement for full-pathway delivery, and hospital-stage support provided when that requirement is not met is recorded as a partial delivery rather than as a protocol deviation.

### 4.4 Constraints on inference

The consent-gated, nonconsecutive referral process limits external validity. Referring clinicians determine which children are offered the pathway, but referrals, preliminary screening, and reasons for non-offer were not recorded. Two of eight caregivers declined, both citing organizational burden; this proportion is conditioned on clinician-selected offering to families judged receptive and therefore does not estimate refusal under consecutive offering, although it identifies caregiver burden as an observed constraint on the home-based component. Consent, eligibility, readiness, venous-access completion, examination completion, partial delivery, and full-pathway completion are distinct process states and should be reported separately. Three of the four CT examinations completed without sedation followed partial rather than full delivery, showing only that awake acquisition can occur through multiple routes. Isolating any contribution of Euterpe-RAD requires consecutive offering and a prospective comparator.

### 4.5 Implications and future directions

#### 4.5.1 Institutional improvement targets

The 2026 Good Practice submission specified an institutional clinical objective of avoiding sedation or general anesthesia during the examination whenever clinically and safely feasible, rather than targeting a predefined percentage reduction. The submission also identified process-improvement aims, including fewer motion-related sequence repetitions, a shorter door-in-to-door-out interval, improved image quality, operator-rated cooperation, caregiver satisfaction, and home adherence. These aims belong to institutional quality-improvement governance. Although some measures may also be evaluated prospectively, they are not research hypotheses, prespecified study thresholds, or evidence of effect; no corresponding value is reported here.

#### 4.5.2 Planned prospective evaluation

A protocol is being prepared for research ethics review. The proposed primary process outcome is completion of the scheduled examination under pathway conditions. Proposed secondary outcomes are sedation-related measures, diagnostic image quality assessed by blinded review, the door-in-to-door-out interval, and child and family procedural experience. Age-appropriate validated measures will be prespecified for cooperation and child- and family-reported procedural experience. Prospective safety monitoring will include systematic recording of headset exposure duration and prespecified exposure-related symptoms or adverse events, together with instrumental verification of ear-level acoustic output under the configured delivery conditions. A concurrently recruited comparison group receiving standard preparation is planned, drawn from the same age band and examination category; allocation and recruitment sequence will be specified in the protocol. These outcomes will be considered prespecified only after the protocol and statistical analysis plan are dated, approved, and prospectively registered. The statistical analysis plan will define the sample-size rationale, analysis populations, handling of missing data and attrition, and the planned estimation and comparison procedures before enrolment begins.

Two denominators will be reported: an implementation denominator fixed at the offer and an analytic denominator fixed at consent, with attrition at each subsequent gate reported separately. The pathway will be offered consecutively to eligible scheduled children, and reasons for non-offer, refusal, exclusion, discontinuation, and incomplete delivery will be recorded. Joint assessment of implementation and effectiveness may warrant a hybrid design (28), but that classification requires an *a priori* protocol. The institutional improvement program will continue independently of the research evaluation.

#### 4.5.3 Headset use below the manufacturer's recommended age

The tolerability data cited in Module 3 (20, 21) narrow, without resolving, the question of headset use below the manufacturer's recommended consumer age. A different headset model was tested in children aged 4–10 years across two consecutive 30-minute recreational sessions, with no measurable change in visuomotor function, postural stability, or vestibulo-ocular adaptation (20). The age band overlaps with the Euterpe-RAD population, but the device, task, and setting do not. A subsequent perspective by Tychsen and Thio, which also discussed this pediatric exposure cohort, reported no cases of photosensitive seizures associated with headset viewing (29). The protocol does not rely on this evidence to establish the safety of headset exposure in Euterpe-RAD; photosensitivity is instead addressed upstream through pre-exposure screening and exclusion when known or suspected photosensitivity is identified, before any device exposure. The pediatric virtual-reality safety literature in children under 14 years remains limited to short, supervised exposures, with transient cybersickness and amblyopia-related diplopia among the principal recurring findings (21). A retrospective review of 213 children aged 6–18 years reported infrequent, self-limited adverse events under structured clinical supervision (dizziness 0.5%, nausea 0.5%, increased anxiety 3.8%) across perioperative and other clinical settings (30). That review had no control group and no standardized method for ascertaining adverse events. None of these sources establishes exposure parameters for the specific headset, age range, content, and procedural conditions used in Euterpe-RAD. The preprocedural session is already bounded, at 5–15 minutes, by the entry criteria specified in Module 3. The duration of this preprocedural session is recorded separately from any subsequent continuation of headset exposure through cannulation, contrast administration, and acquisition; total headset exposure from fitting to removal is also documented. Supplementary Material 1 sets no threshold for this longer, cumulative interval. Current mitigation comprises the stop criteria and continuous clinical supervision already specified in Module 3, consistent with the structured clinical supervision reported in the retrospective review cited above (30). No exposure duration reported in the available pediatric literature is treated here as a validated device-specific safety threshold. The formal institutional risk assessment, still pending at the census date, will need to evaluate the specific device, define an appropriate maximum exposure duration for the intended pediatric and procedural conditions, and determine whether the existing stop criteria remain adequate before headset use is extended beyond the present deployment.

## 5 Limitations

This community case study describes implementation at one institution; it does not evaluate effectiveness or causality. Only one child completed the full pathway, no MRI examination was delivered, and transferability remains untested. Pathway offering was clinician-selected and nonconsecutive, and no comparator or effectiveness analysis was performed, limiting external validity and preventing attribution of observed procedural outcomes to Euterpe-RAD. Only an approximate service-volume denominator was available, offer-level screening denominators were not recoverable, and aggregate fidelity and adherence values could not be estimated from a single complete delivery. The cooperation measure was not in routine use, inter-coder agreement was not estimated, ear-level acoustic output was not instrumentally verified during the reported deployment, and headset use below the manufacturer's recommended consumer age had not undergone a formal institutional risk assessment by the census date. These limitations preclude claims of clinical benefit, acceptability, a sedation-sparing effect, or replicability and do not establish device-specific safety.

## 6 Conclusion

By the census date, one child had completed the five-module Euterpe-RAD pathway during contrast-enhanced CT without sedation or modification of acquisition or contrast protocols; the MRI branch remained undelivered. The contribution of this case study is an operational specification for sustaining an autobiographically anchored multisensory context through content identity, transition coverage, and stable caregiver and therapeutic roles. This single delivery does not establish effectiveness, acceptability, transferability, or a sedation-sparing effect. Effects on sedation use, diagnostic image quality, workflow, and procedural experience require prospective comparative evaluation.

## Data Availability

The original contributions presented in the study are included in the article/Supplementary Material, further inquiries can be directed to the corresponding author.
