# Supplementary material for: A person-centered continuity pathway for pediatric contrast-enhanced imaging: sustaining one multisensory context across home, hospital, and scanner transitions
Source: Front Health Serv. 2026 Sep 30;6:1940926. doi: 10.3389/frhs.2026.1940926 (PMC13662128; doi:10.3389/frhs.2026.1940926)
Supplement: Supplementary file 1 [file Datasheet1.pdf]

Supplementary Material

Structured procedural checklist, version record, and acoustic specification (Euterpe-RAD)

This file provides a structured procedural checklist covering identification, rationale, materials, procedures, provider, delivery mode, setting, schedule, tailoring, modifications and fidelity, together with the fidelity indicator definitions, the acoustic specification and the version record of the pathway. Domains follow the Template for Intervention Description and Replication (TIDieR). The music-based component is additionally cross-mapped to the Reporting Guidelines for Music-Based Interventions (RG-MBI) in Table S2. Table S3 records protocol versions and modifications. Table S4 provides a selective cross-reference to potentially applicable SQUIRE 2.0 items; it is a statement of scope and not a claim of adherence. The file contains no patient- or family-identifying information.

Table S1. Intervention description (TIDieR domains).

Domain	Reporting
1. Identification	Euterpe-RAD: consent-gated, five-module institutional pathway sustaining a single autobiographically anchored multisensory context across pediatric contrast-enhanced CT and MRI, including venous-access support (ages 4–10 years); included in the OPBG 2026 institutional Good Practice program, list circulated by the Medical Directorate (note no. 661/26 of 20 March 2026).
2. Procedural rationale	Existing nonpharmacological interventions act on temporally circumscribed segments of the procedural pathway and do not preserve continuity across the home–hospital–scanner transition (Introduction). The rationale is operational, not mechanistic. Continuity is defined by three verifiable conditions: content identity across the modules that require it; coverage of four predefined transitions, meaning the planned re-establishment of at least one element of the personalized frame at each change of setting, activity or personnel, without any implication of continuous stimulation; and stability of the caregiver role together with continuity of the therapeutic role and delivery plan.
3. Materials	Recorded family voices; personalized therapeutic composition (approximately 3–4 min); autobiographical images and video; environmental projection surfaces appropriate to the delivery environment; standalone VR headset (Meta Quest 3); an appropriately labeled MR Safe or MR Conditional pneumatic headphone system; an in-bore visual channel controlled from outside the scanner room; institutional tele-delivery platform; and traceable content archives. The MRI delivery equipment is specified functionally and not as a verified scanner-specific installation.

4. Procedures	Full-pathway delivery requires a child scheduled for a contrast-enhanced examination, with venous access planned; signed caregiver consent; documented electronic authorization from the head of the referring clinical unit for the music therapist to contact the family; and an interval to the scheduled examination sufficient for the 5–7-day home-familiarization window. When that interval is unavailable, Module 1 does not begin. Any hospital-stage music-therapy support is recorded as a partial delivery rather than as a protocol deviation or five-module completion. Five sequential modules: (1) tele-anamnesis, eligibility confirmation, collection of authorized material, production of the personalized content, home familiarization and verification of the content that will be used in the immersive room; (2) caregiver briefing in the anteroom on the day of the examination, with procedural verification and preparation for co-regulation; (3) preprocedural immersion in the immersive room, with the headset fitted before cannulation in the CT branch; (4) cannulation, contrast administration and acquisition, in the immersive room in the CT branch without transfer of the child, and after transfer to the magnet room in the MRI branch; (5) de-immersion and closure. Gating: caregiver consent precedes Module 1; eligibility is confirmed within Module 1 during the tele-anamnesis with which it opens; an operator-rated readiness threshold precedes cannulation and acquisition. Venous-access completion is a distinct process variable, separate from the readiness threshold. Non-consenting or ineligible children, and those who do not meet the readiness criterion, follow the standard institutional pathway.
5. Provider	Multidisciplinary team. Tele-anamnesis, neuropsychological screening and production of the personalized therapeutic composition: neurorehabilitation team. Module 1 home familiarization: caregiver, under the team's remote supervision. Modules 2–5: qualified music therapist in coordination with nursing and radiology staff; after preparation the therapist leaves the room and tolerability is monitored from the control room by radiography and nursing staff. Caregiver in-room presence is governed by modality-specific safety requirements and by departmental practice: permitted in CT under radiation-protection provisions; in MRI, determined by local MR safety screening and departmental rule. A psychotherapist is present across all five modules, from the opening tele-anamnesis to postprocedural closure.
6. Mode of delivery	Module 1: caregiver-mediated home delivery through the institutional tele-delivery platform under remote supervision, with caregiver video feedback. Modules 2–5: face-to-face. CT: environmental projection and supervised headset delivery in the immersive/acquisition room. MRI design specification: appropriately labeled MR Safe or MR Conditional pneumatic headphones and an in-bore visual channel; the standalone headset remains outside the MR controlled access area.
7. Setting	Home (Module 1, days −7 to −1); hospital anteroom (Module 2, day 0, briefing only, no clinical act and no content delivered to the child); immersive room (Modules 3 and 4, day 0), which in the CT branch is the

	acquisition room configured as the immersive environment; magnet room (MRI branch only, after venous access); immersive room or recovery area (Module 5).
8. Schedule and dose	Module 1: 5–7 days (approximately days −7 to −1), 2–3 administrations/day of the complete audiovisual sequence. Module 2: day 0, before entry to the immersive room. Module 3: day 0, preprocedural session 5–15 min. Module 4: CT branch, headset retained during cannulation, contrast administration and acquisition where compatible, with total headset exposure recorded from fitting to removal and the 5–15-min Module 3 interval distinguished from any continuation through cannulation, contrast administration, positioning, or acquisition; MRI branch, venous access in the immersive room and continuity sustained through appropriately labeled MR Safe or MR Conditional audio and in-bore visual relay for the duration of the examination. Module 5: immediately post-acquisition; duration not separately quantified.
9. Tailoring	Neurobehavioral stratification: intensified Module 1 delivered at the upper (7-day) end of the window, with additional support, for children with documented attentional or emotional-regulation vulnerability, applied as a modular adjustment and not as an exclusion criterion.
10. Modifications	Reported in Table S3, which records the change from Euterpe-RAD v1.0 (institutional specification submitted to the 2026 Good Practice program) to Euterpe-RAD v2.0 (consolidated specification described in the main text).
11. Fidelity, planned	See Fidelity indicator definitions below. Entry to Module 4 is currently gated by clinical judgment and not by a scored instrument; for prospective research evaluation, an age-appropriate validated cooperation measure will be prespecified and is not yet in routine use.
12. Fidelity, actual	Per-child module-checklist documentation was completed for each pathway module actually delivered, through the point at which the child left the pathway. Hospital-stage support delivered without Module 1 was recorded separately as a partial delivery and was not treated as a five-module completion. In the single complete pathway, all four predefined transitions were documented as covered, with no content deviation recorded. Aggregate values are not derivable from one complete delivery: home adherence, content fidelity, and inter-coder agreement are reserved for prospective evaluation. Descriptive service-activity counts in the main manuscript are not fidelity estimates.

Table S2. Music-based component (RG-MBI cross-mapping).

RG-MBI component	Reporting
1. Brief name	Personalized therapeutic composition within the Euterpe method [11].
2. Theory / scientific rationale	Operational, not mechanistic: continuity of a single autobiographically anchored context across the procedural pathway; no neurocognitive mechanism is claimed.

3a. Music selection	Family voices recorded in a guided narrative; autobiographical visual content selected with the child and family; musical preferences collected during the tele-anamnesis.
3b. Music characteristics	Recorded family voices set within a rhythmic-melodic structure, of approximately 3–4 min. Tempo range 60–108 bpm. Dynamics restricted to the mezzo-piano to mezzo-forte range and delivered without crescendos, which is an acoustic and not an aesthetic constraint (see Acoustic specification below), since a compressed dynamic range holds the margin above ambient approximately constant.
3c. Delivery method	Recorded. Home: tele-delivery platform, composition, images and video presented simultaneously. CT branch: environmental projection and supervised headset delivery in the immersive room, sustained through cannulation, contrast administration and acquisition. MRI branch: appropriately labeled MR Safe or MR Conditional pneumatic headphones and scanner-integrated in-bore relay; no headset in the MR controlled access zone.
3d. Materials	As Table S1, Domain 3.
3e. Intervention strategy	Receptive at home; interactive within the headset session and during the CT procedure where the headset is retained; combined with environmental projection; a single sustained context rather than discrete episodes.
4. Interventionist	Qualified music therapist working with the neurorehabilitation team, psychotherapist, nursing staff, and radiology staff; professional roles and setting-specific responsibilities are detailed in Table S1, Domain 5.
5. Individual or group	Individual (one child), with the caregiver as co-regulator where permitted by modality-specific safety constraints.
6. Setting	As Table S1, Domain 7.
7. Delivery schedule	As Table S1, Domain 8.
8. Treatment fidelity	Planned: adherence logging (at least 80% target), Module 1; ICC at least 0.80 for structured coding. Actual: module-checklist documentation was completed for each module actually delivered, through the point at which the child left the pathway; descriptive findings are reported only for the single complete pathway, and no aggregate fidelity estimate was derived.

Acoustic specification

The controlled parameter is the level difference between the delivered content and the measured ambient noise of the delivery setting, expressed in dB(A) at the child's head position, and not an absolute sound pressure level. Specifying a margin rather than an absolute level permits comparison across settings with different noise floors; the margin must be set instrumentally.

As a prospective operational specification, delivery in the immersive room is to be set at approximately 10–15 dB(A) above the ambient equivalent continuous level measured at the child's

head position and is not to exceed 75 dB(A) irrespective of ambient level. Acoustic output at the child's head position under the configured delivery conditions was not instrumentally verified during the deployment described in the main text, and no claim is made that delivery achieved a prespecified margin or ceiling. The margin is taken by analogy from studies of speech recognition in noise, in which school-age children require a signal-to-noise ratio 5–10 dB more favorable than adults, and at least 10 dB is required for near-ceiling recognition under low reverberation [15,16]; the analogy concerns intelligibility of the spoken component rather than audibility of the musical material, and the transposition to a procedural setting has not been validated. The upper limit follows the more conservative safe-listening values recommended for children [17]. Ambient level is to be measured before delivery with a calibrated class 2 integrating sound level meter positioned at the child's head, over an interval of at least 60 s, by the delivering therapist, and re-verified whenever the delivery room or equipment changes. Instrumental verification of ambient and delivered levels at the child's head position under the configured delivery conditions is a requirement of the prospective protocol and was not performed during the deployment described.

Formal statement of the acoustic specification

The controlled quantity is the margin $\Delta L = L_{Aeq,signal} - L_{Aeq,ambient}$, both terms expressed in dB(A) at the child's head position and obtained with the same frequency weighting and the same integration interval. The prospective specification is $10 \leq \Delta L \leq 15$ dB, with $L_{Aeq,signal}$ not exceeding 75 dB(A).

Two consequences follow, and both are operational rather than theoretical. First, a meter reading taken during delivery registers the energetic sum of signal and ambient rather than the signal alone, so the signal level is recovered as $L_{signal} = 10 \log_{10} (10^{(L_{total}/10)} - 10^{(L_{ambient}/10)})$. The ambient level must therefore be measured before delivery begins, and the correction applied to any verification reading. Second, in a setting whose ambient level exceeds approximately 60–65 dB(A), the specified margin and the 75 dB(A) ceiling cannot both be satisfied; in that case the ceiling prevails, the margin is reported as not achieved, and the discrepancy is recorded. No measured margin is reported for this deployment; the 10–15-dB specification remains transposed by analogy from speech-in-noise studies and has not been validated for pediatric procedural settings.

Within the magnetic-resonance environment this logic does not apply and is not attempted. Acquisition noise reaches levels at which audibility above ambient could not be obtained within any safe delivery limit, and hearing protection is mandatory [18]. Content is delivered beneath that protection through appropriately labeled MR Safe or MR Conditional pneumatic headphones, whose passive attenuation determines the level at the ear. The objective in this segment is the presence of familiar content, not its dominance over scanner noise, and continuity is carried principally by the in-bore visual relay. Delivery level would be verified at installation and would not be raised during acquisition.

Fidelity indicator definitions

Home adherence. Numerator: home administrations completed and logged. Denominator: home administrations scheduled for that child. Unit of observation: child. Source: tele-delivery platform log. Window: Module 1. Threshold: at least 80% per child, applied individually and reported in aggregate. Missing logs are counted as not completed.

Content fidelity. Numerator: personalized elements traceable to an item of the sensory profile or to authorized material. Denominator: personalized elements produced for that child. Expressed as a percentage. Unit of observation: element within child. Source: content archive of the method.

Window: from completion of the tele-anamnesis to the freezing of the personalized material at the end of production. Assessor: a member of the neurorehabilitation team not involved in producing the material. Threshold: all elements traceable. An element whose derivation is not documented counts as not traceable, and an element replaced after freezing is recorded as a deviation with its reason.

Inter-coder agreement. Object: structured coding of the personalized material within the parent method. Coders: two, trained on the coding manual of the parent method. Source: content archive. Subsample: at least 20% of coded material and not fewer than ten records, drawn at random within the reporting period. Window: computed once per reporting period of the prospective evaluation. Criterion: ICC of at least 0.80, two-way mixed model, absolute agreement, single measures, reported with its confidence interval. Values below the proposed criterion would trigger retraining and recoding of the full set for that reporting period. The ICC threshold is proposed for the prospective evaluation and will be considered prespecified only after the protocol and statistical analysis plan are dated, approved, and prospectively registered; inter-coder agreement was not estimated for the present deployment.

Continuity fidelity. Two ratios. Content identity: modules delivered with the prespecified identical content set over modules requiring identical content, where a permitted adaptation is a change of delivery channel only and any change of content is recorded as a deviation. Transition coverage: predefined transitions covered over transitions requiring coverage, expressed as a percentage. Unit of observation: transition within procedure. Source: module delivery checklist. Window: from the start of Module 1 to the end of Module 5 for that child. Threshold: all four transitions covered. The four predefined transitions are home familiarization to hospital arrival, anteroom briefing to immersive room, preprocedural immersion to cannulation and acquisition, and acquisition to postprocedural closure. A transition is covered when at least one prespecified element of the frame is intentionally re-established and recorded on the delivery checklist. It is classified as not covered, and as a protocol deviation, when no intended element is available or re-establishment is undocumented. Continuous stimulation during travel or between scheduled sessions is not required. At the first transition, coverage is documented as caregiver-mediated re-establishment; delivery of content to the child is not required at Module 2. Relational stability is assessed as stability of the caregiver role and continuity of the therapeutic role and delivery plan; substitution of the delivering therapist is recorded as a deviation and does not by itself constitute a failure of coverage, and the departure of the therapist from the room after preparation is not a failure of coverage provided the programmed content continues. Where documentation is missing, the transition counts as not covered. Relational stability is not a separate indicator: it is a descriptive requirement of the pathway, and its failure is captured as a recorded deviation rather than as a ratio. Recording is the responsibility of the delivering music therapist on the module checklist, which was in use throughout the deployment described; the thresholds and aggregate ratios defined above apply to the prospective evaluation.

Time to eligibility confirmation. Interval in days from caregiver consent to confirmation of eligibility during the tele-anamnesis that opens Module 1. Unit of observation: child. Source: tele-delivery platform log and neurorehabilitation record. Reported as a descriptive interval; no threshold is set.

Exposure duration. Interval from fitting of the headset to its removal, recorded per procedure, distinguishing the preprocedural session from any continuation through cannulation, contrast

administration and acquisition. Unit of observation: procedure. Source: module checklist. Reported descriptively; no threshold is set.

Prospective safety monitoring. The prospective protocol will prespecify recording and classification of exposure-related symptoms or adverse events using the Module 3 stop-criteria documentation and Module 5 immediate post-exposure observation, together with total headset exposure duration. No aggregate safety estimate is derived from the reported deployment.

The thresholds and aggregate ratios defined above apply to the prospective evaluation. During the reported deployment, module-checklist documentation was completed for each module actually delivered. Only the descriptive checklist findings for the single complete pathway are reported; no aggregate fidelity estimate was derived.

Table S3. Version record of the pathway specification (TIDieR Domain 10).
Version identifiers: Euterpe-RAD v1.0, the institutional specification submitted to the 2026 Good Practice program in February 2026; Euterpe-RAD v2.0, the consolidated specification, effective from April 2026 and approved by the participating units under the institutional Good Practice governance. Operational delivery within the pediatric imaging service began under v2.0, which was in effect throughout the interval covered by the census date; the recorded pathway encounters—one full five-module completion, three partial deliveries, one safety exclusion, and one procedural discontinuation—therefore fall under v2.0. In three consenting children, the interval between referring-unit authorization and the scheduled examination was shorter than the home-familiarization window, so Module 1 was not delivered. These children received hospital-stage music-therapy support only and are recorded as partial deliveries constrained by lead time, not as protocol deviations or five-module completions.

Element	v1.0, institutional specification	v2.0, effective April 2026
Home-based encoding window	3–5 days, 2–3 administrations/day	5–7 days (days −7 to −1), 2–3 administrations/day. Design rationale: the window was extended to separate the time required to produce and deliver the personalized material from the exposure required for it to become familiar, which is the condition of content identity at the hospital transition.
Preprocedural immersion	Environmental projection and audiovisual delivery in a multisensory room	Addition of a 5–15 min standalone VR session in the immersive room, outside the MR controlled access zone. Design rationale: environmental projection reconstructs the personalized context but does not screen the child from the procedural environment; the headset was introduced to occlude that environment during the immediately preprocedural window without altering content, which strengthens transition coverage at the point where the design judged it weakest. No measurement of arousal was made.
Delivery setting on day 0	Multisensory room distinct from the procedure environment	Immersive room, which in the CT branch is the acquisition room configured as the immersive environment, so that preprocedural immersion, cannulation, contrast administration and acquisition occur without transfer of the

		child; caregiver briefing relocated to the anteroom on the day of the examination. Design rationale: transfer between spaces was considered the point at which the personalized frame was most vulnerable to interruption.
Postprocedural closure	Not specified as a distinct step	Formalized as Module 5. Design rationale: to avoid abrupt withdrawal of the immersive context at the end of acquisition and to provide structured re-orientation. No outcome was measured before or after the change.
Age window	Not restricted; indicators included neonates and infants	Restricted to 4–10 years. Design rationale: an institutional operating criterion, since Module 1 requires comprehension of a guided narrative and sustained receptive engagement; above ten years the marginal value declines in children who complete awake acquisition with standard preparation (main text, Section 2.2).
Modality branches and indications	Initial phase limited to cardiac MR and cardiac CT for organizational reasons	Authorized across cardiac, body and neuroradiological indications; initial delivery involved cardiac contrast-enhanced CT, and the MRI branch remained undelivered by the census date. Design rationale: the initial limitation was organizational rather than clinical, and branch-specific safety constraints depend on modality and not on indication.

No further versioned change to the pathway specification is described for the deployment reported here. Aggregate fidelity data and any subsequent protocol modification will be documented in the planned prospective evaluation.

Selective cross-reference to potentially applicable SQUIRE 2.0 items

Table S4. Selective cross-reference to potentially applicable SQUIRE 2.0 items. The primary editorial standard for this article is the Frontiers community case study format; TIDieR provides the structure for describing the intervention (Table S1); individual SQUIRE items, interpreted through the accompanying explanation and elaboration document, were used only as an internal descriptive aid. No adherence, conformity or combined reporting is claimed.

SQUIRE 2.0 item	Scope in this article
1. Title	Limited relevance. Title follows the community case study format; the article is not labeled a quality-improvement study.
2. Abstract	Journal format. No SQUIRE statement is made in the abstract.
3. Problem description	Used as a descriptive aid. Introduction, paragraphs 1–2.
4. Available knowledge	Used as a descriptive aid. Introduction, paragraph 2.
5. Rationale	Used with caution. Operational rationale in the Introduction; continuity stated as an untested design hypothesis.

6. Specific aims	Used with caution. The manuscript reports institutional improvement aims without presenting predefined percentage reductions; these aims remain distinct from the proposed outcomes of the future study.
7. Context	Used as a descriptive aid. Sections 2.1, 2.2 and 3.2.
8. Intervention(s)	Used as a descriptive aid, structured primarily by TIDieR. Sections 3.2 and 3.3; Table 1; Tables S1–S3.
9. Study of the intervention	Outside the current report. No study of the intervention was performed; the audit plan is described in Section 2.1 and the future protocol in Section 4.5.2.
10. Measures	Limited descriptive relevance. Operational definitions are provided in Table 1 and in the fidelity indicator definitions above. The current report includes only descriptive checklist findings for the single complete pathway; no aggregate fidelity or outcome estimate is analyzed. The prospective protocol will prespecify age-appropriate validated measures for cooperation and child- and family-reported procedural experience and will include instrumental acoustic verification under configured delivery conditions (Section 4.5.2).
11. Analysis	Outside the current report; the prospective statistical analysis plan will prespecify the sample-size rationale, analysis populations, handling of missing data and attrition, and planned estimation and comparison procedures before enrolment begins.
12. Ethical considerations	Used as a descriptive aid. Governance and data protection in Section 2.1; Ethics statement; Conflict of Interest statement; future ethics review in Section 4.5.2.
13. Results	Limited descriptive relevance: implementation history, version record, and delivery counts only. No process or outcome effect and no association is reported.
14. Summary	Used as a descriptive aid. Section 4.2 and Conclusion, answering the implementation question only.
15. Interpretation	Limited relevance. Sections 4.1–4.3 report lessons learned; no causal association is drawn.
16. Limitations	Used as a descriptive aid. Sections 4.4 and 5: selection, missing counts, MRI branch not delivered, no comparator, aggregate fidelity not estimable from a single complete delivery; acoustic output not instrumentally verified and no formal institutional headset risk assessment completed by the census date.
17. Conclusions	Used with caution. Section 6: local delivery and next steps; neither sustainability nor spread is demonstrated.
18. Funding	Used as a descriptive aid. Funding statement.
Abbreviations: CT, computed tomography; dB(A), A-weighted decibels; ICC, intraclass correlation coefficient; MR, magnetic resonance; MRI, magnetic resonance imaging; RG-MBI, Reporting Guidelines for Music-Based Interventions; SQUIRE, Standards for QUality Improvement Reporting Excellence; TIDieR, Template for Intervention Description and Replication; VR, virtual reality.	
